# Fatigue-Associated Alterations in Gut Microbiota, Mitochondrial Energy Metabolism, and Immune Function in Mice: Implications for Future Nutrition Studies

**DOI:** 10.3390/nu18122031

**Published:** 2026-06-22

**Authors:** Menghui She, Huiyi Peng, Qin Liu, Zhoujin Tan

**Affiliations:** 1 School of Traditional Chinese Medicine, Hunan University of Chinese Medicine, Changsha 410208, China; 15200837048@163.com (M.S.); anapenghuiyi@163.com (H.P.); liuqin998@163.com (Q.L.); 2 Hunan Key Laboratory of Traditional Chinese Medicine Prescription and Syndromes Translational Medicine, Changsha 410208, China

**Keywords:** fatigue, immune function, energy metabolism, gut microbiota

## Abstract

**Background:** This study investigated the relationships among mitochondrial energy metabolism, immune function, and gut microbiota in mice under a fatigued state, providing preliminary evidence for future nutrition-related mechanistic and intervention studies. **Methods:** Mice were adaptively fed for 4 days and then randomly divided into a normal control group (NC) and a fatigue model group (NM). Immune organ indices, serum IgG levels, thigh muscle ATP content, mitochondrial respiratory chain complex I–IV activities, and gut microbiota composition were assessed using enzyme-linked immunosorbent assay (ELISA), microplate assays, and 16S rRNA gene sequencing. **Results:** Compared with the NC, the NM showed a significantly reduced spleen index, serum IgG levels, mitochondrial respiratory chain complex I, III, and IV activities, along with reduced ATP content. Regarding gut microbiota, mice in the NM exhibited disordered intestinal villus arrangement, inflammatory cell infiltration in the crypts and muscular layers, and markedly reduced intestinal microbial activity as well as protease and sucrase activities. 16S rRNA sequencing revealed fewer ASVs in the NM, with enrichment of *Lactobacillaceae*, *Limosilactobacillus*, and *Ligilactobacillus*, whereas the NC was characterized by *Borkfalkiaceae* and *Borkfalkia*. Linear discriminant analysis effect size (LEfSe) analysis identified *Lactobacillaceae*, *Firmicutes_D*, and *Lactobacillales* as characteristic taxa of the NM. Kyoto Encyclopedia of Genes and Genomes (KEGG) prediction indicated that fatigue-associated microbial functions were mainly related to *carbohydrate*, *amino acid*, and *lipid metabolism*. Correlation and RDA analyses further suggested that alterations in gut microbiota structure were closely associated with mitochondrial energy-related indicators and immune-related parameters. **Conclusions:** Fatigue was associated with alterations in energy metabolism, immune function, and gut microecology in mice. The “gut microbiota–energy metabolism–immunity” framework may represent a potential association-based framework and provides biological information to support future nutrition-related intervention studies.

## 1. Introduction

Fatigue is a common yet complex, multidimensional symptom that encompasses physical, psychological, cognitive, emotional, and motivational fatigue, with physical fatigue being the most extensively documented dimension [1]. To date, a universal definition of fatigue has not been established. It is generally characterized by a persistent or recurrent sense of exhaustion, a decline in physical or mental performance, and reduced activity capacity, which is not fully resolved by rest [2]. The etiology of fatigue is multifactorial and closely associated with lifestyle, disease, excessive physical exertion, infections, and other factors [3,4].

Its underlying links may involve dysregulation of the central nervous system, mitochondrial dysfunction, oxidative stress, inflammatory responses, fluctuations in immune function, and gut microbiota imbalance [5,6,7]. Furthermore, fatigue is a global health concern. A meta-analysis indicates that approximately 20% of the global population experiences fatigue [8]. Its prevalence also correlates with demographic factors such as sex, age, and occupation, being higher in adults than in minors and in females than in males [8], with rates in certain professional populations reaching 42.3% [9]. Particularly at risk are individuals exposed to prolonged high workloads, shift work, sleep deprivation, or high psychological stress, such as healthcare workers [10].

To investigate the biological effects of fatigue, an appropriate animal model is required to mimic sustained physical exertion. In the present study, fatigue was induced by prolonged standing, which simulates a subhealthy condition characterized by functional imbalance resulting from sustained physical load, rather than representing a specific pathological disorder [11]. If fatigue becomes chronic and is not effectively managed, it can impair daily life, work, and learning [12] and may be related to damage across multiple physiological systems, including anxiety, depression, insomnia, and gastrointestinal disorders [13]. The role of gut microbiota dysbiosis in fatigue has emerged as a prominent area of research in recent years. A review of studies over the past decade reveals that fatigue induces alterations in gut microbes, including changes in microbial diversity and community structure [14]. Network pharmacology studies further suggest that modulating the gut microbiome may be a viable strategy for alleviating fatigue [15]. Other studies have reported that fatigue is associated with mitochondrial abnormalities [16], and fatigue can also affect the stress responses of immune cells [17]. Moreover, the gut microbiome is involved in numerous physiological processes, including energy metabolism and immunity [18]. It can regulate lipid and energy metabolism through short-chain fatty acids and bile acids [19,20], and it also influences immune system development and maturation, thereby regulating immune function [21].

Although increasing evidence suggests that fatigue is associated with mitochondrial dysfunction, immune dysregulation, and gut microbial imbalance, most previous studies have examined these mechanisms separately. However, it remains unclear whether fatigue induced by sustained physical exertion simultaneously disrupts gut microbiota composition, mitochondrial respiratory chain activity, ATP production, and immune indicators. Consequently, this research conducts a systematic investigation of fatigue from the three perspectives of energy metabolism, immune modulation, and gut microecology, aiming for a comprehensive understanding of the fatigue phenomenon. Specifically, we assessed energy metabolism by measuring the activities of mitochondrial respiratory chain complexes I–IV and ATP levels in the thigh muscles of mice. Immune regulation was evaluated by analyzing serum IgG levels and the thymus and spleen indices. Gut microbiota composition was assessed using 16S rRNA gene sequencing. Because gut microbiota composition, immune status, and energy metabolism are closely linked to host nutritional status, this study may provide preliminary biological evidence for future nutrition-related research aimed at understanding physiological changes under fatigued conditions.

## 2. Materials and Methods

### 2.1. Experimental Animals and Housing Environment

Male KM mice [22] (SPF-grade, 4 weeks old, body mass 20 ± 2 g) were purchased from Hunan Slake Jingda Experimental Animal Co., Ltd (Changsha, China). [License No.: SCXK (Xiang) 2021-0002]. The mice were housed at the Experimental Animal Centre of Hunan University of Chinese Medicine [License No.: SYXK (Xiang) 2024−0014]. The animals were not genetically modified, and no previous procedures were performed before this study. The experimental protocol was approved by the Institutional Animal Care and Use Committee of Hunan University of Chinese Medicine (Changsha, China), ensuring compliance with ethical guidelines for animal research [Ethics No.: HNUCM21-2510-12].

The mice were maintained under strictly controlled conditions: a room temperature of 23–25 °C, relative humidity of 50–70%, and a 12 h light/dark cycle. They were provided with ad libitum access to food and water. The standard chow was supplied by the Experimental Animal Centre of Hunan University of Chinese Medicine, manufactured by Beijing Huafukang Biotechnology Co., Ltd. (Beijing, China) [Feed License No.: Jing Shi Zheng (2024) 06076].

### 2.2. Assay Kits

Mitochondrial respiratory chain complex I/NADH-coenzyme Q reductase assay kit for mice (Jiangsu Edison Biotechnology Co., Ltd., Yancheng, China, Cat No.: ADS-W-FM006-48); Mitochondrial respiratory chain complex II/succinate-coenzyme Q reductase assay kit for mice (Jiangsu Edison Biotechnology Co., Ltd., Cat No.: ADS-W-X002-48); Mitochondrial respiratory chain complex III/CoQ-cytochrome C reductase assay kit for mice (Jiangsu Edison Biotechnology Co., Ltd., Cat No.: ADS-W-X012-48); Mitochondrial respiratory chain complex IV/cytochrome C oxidase assay kit for mice (Jiangsu Edison Biotechnology Co., Ltd., Cat No.: ADS-W-X010); Mouse adenosine triphosphate (ATP) ELISA kit (Jiangsu Jingmei Biotechnology Co., Ltd., Yancheng, China, Cat No. JM-11362M2); Mouse IgG ELISA kit (Shanghai Zhuocai Biotechnology Co., Ltd., Shanghai, China, Cat No. ZC-38497W).

### 2.3. Experimental Instruments

Autoclave (Model SQ510C, Yamato Scientific Co., Ltd., Tokyo, Japan); Electric blast drying oven (GZX-9070MBE, Shanghai Boxun Medical Biological Instrument Corp., Shanghai, China); Electric constant temperature incubator (Model 303-4B, Shaoxing Shangcheng Instrument Manufacturing Co., Ltd., Shaoxing, China); High-speed refrigerated centrifuge (5810R, Eppendorf, Hamburg, Germany); Ultrapure water system (ELGA, High Wycombe, UK); Vortex mixer (VORTEX 5, Kylin-Bell Lab Instruments Co., Ltd., Nantong, China); UV-Vis spectrophotometer (Nano drop 2000/2000C, Thermo Scientific, Waltham, MA, USA); Multifunction microplate reader (SPARK, Tecan Group Ltd., Männedorf, Switzerland); Water bath (SSW-420, Shanghai Boxun Medical Biological Instrument Corp., Shanghai, China); Rotarod fatigue tester (XR-6C, Shanghai Xinruan Information Technology Co., Ltd., Shanghai, China).

### 2.4. Experimental Grouping and Model Preparation

After a 4-day adaptive feeding, 20 KM mice were assigned to the normal control group (NC) and fatigue model group (NM) using a random number table, with 10 mice in each group. No formal blinding procedure was implemented during the study, considering the nature of the animal modelling procedure and the need to maintain procedural continuity and consistency. Furthermore, no formal a priori sample size calculation was performed, and no animal death or attrition occurred during the experiment.

The fatigue model was induced by subjecting NM mice to a forced standing on a small platform in water for a fixed duration of 4 h per day (from 8:30 a.m. to 12:30 p.m.) for 14 consecutive days, thereby establishing a model of fatigue due to prolonged standing [23,24,25]. Water was used solely to prevent accidental falls, ensuring mice remained standing throughout the 4 h daily sessions. This model simulates sustained physical exertion, a major contributor to fatigue, while minimizing additional stressors.

### 2.5. Index Detection

#### 2.5.1. General Condition of Mice

Throughout the modelling period, the mice’s weight, food and water intake, mental state, activity levels, and physical condition were monitored. Body weight, food intake, and water consumption were specifically recorded on days 1, 5, 9, and 13 of the modelling process. Food and water intake were recorded at the cage level.

#### 2.5.2. Rotarod Fatigue Test

Upon completion of the modelling, five mice from each group were randomly selected for the rotarod fatigue test. Before testing, mice were acclimated to the testing room for 30 min and then adapted to the rotating rod, increasing the speed from 5 rpm to 30 rpm. During the formal test, the rotation speed was set at 30 rpm, and each mouse was tested for a maximum of 10 min. The latency to fall, or the exercise duration on the rotating rod, was recorded as the indicator of motor endurance and fatigue-like performance. After each trial, the apparatus was cleaned to remove feces and urine and sprayed with 75% ethanol to eliminate residual odors. The next trial was started after the ethanol had completely evaporated [26].

#### 2.5.3. Open-Field Test

After modelling, five mice per group were randomly acclimated for 30 min before the open field test using the KSYY-OP-V4.0 system. Each mouse was placed in the arena for 5 min; total distance and speed were automatically recorded. Testing was conducted in the dark. Following each trial, the arena was cleaned with 75% ethanol to prevent cross-contamination and interference among test subjects [27].

#### 2.5.4. Serum IgG Measurement

At the conclusion of the experiment, blood was collected from the retro-orbital sinus of anaesthetized mice, which were subsequently euthanized by cervical dislocation. The blood samples were allowed to clot at room temperature for 3–4 h before being centrifuged at 4 °C and 3000 rpm for 10 min. The resulting supernatant was collected as the serum sample. Serum IgG levels were determined by ELISA using a kit from Shanghai Zhuocai Biological Technology Co., Ltd., and all procedures were performed according to the manufacturer’s instructions.

#### 2.5.5. Organ Index

Mice were euthanized by cervical dislocation under anesthesia. The spleen and thymus were harvested in a sterile setting. Connective tissue was removed, and the organs were blotted dry on filter paper. The organs were weighed, and the organ index was calculated.

Organ Index (%) = Organ Weight (g)/Body Weight (g) × 100%

#### 2.5.6. Mitochondrial Respiratory Chain Complexes I-IV Activity Assay

Immediately under aseptic conditions, thigh muscle tissue was harvested from the mice and flash-frozen at −80 °C. The activities of mitochondrial respiratory chain complexes I-IV were measured using a microplate assay kit from Jiangsu Edison Biotechnology Co., Ltd., following the provided protocol.

#### 2.5.7. ATP Content Assay

Thigh muscle tissue was immediately dissected from the mice under sterile conditions. The ATP content was measured using an ELISA kit from Jiangsu Jingmei Biotechnology Co., Ltd., following the manufacturer’s protocol.

#### 2.5.8. Hematoxylin–Eosin (H&E) Staining

The small intestine was immediately excised under aseptic conditions, connective tissue was removed, and the sample was fixed in 4% paraformaldehyde. Tissue was dehydrated, cleared, and embedded in paraffin for sectioning. After staining with H&E, pathological changes in the intestinal tissue were examined under a microscope.

#### 2.5.9. Measurement of Intestinal Microbial Activity

Under aseptic conditions, the intestinal lumen was gently squeezed using sterilized forceps to collect contents from different segments of the mouse small intestine. The collected contents were transferred into sterile centrifuge tubes containing glass beads. The total weight of each sample was recorded, and the intestinal content was mixed with sterile water at a ratio of 3:50 (*w*/*v*) and vortexed for 30 min. The mixture was then centrifuged at 3000 rpm for 15 min at 4 °C, and the supernatant was collected as the crude enzyme solution [28].

Intestinal microbial activity was assessed using the fluorescein diacetate (FDA) hydrolysis method. Blank control group: acetone was added before incubation to terminate enzymatic hydrolysis, and the mixture contained 2 mL of FDA reaction solution, 2 mL of acetone, and 10 μL of crude enzyme solution. Experimental group: 2 mL of FDA reaction solution and 10 μL of crude enzyme solution were incubated at 24 °C for 90 min, followed by the addition of 2 mL of acetone to terminate the reaction. The absorbance was measured at 490 nm using a UV–visible spectrophotometer. Each sample was assayed in triplicate, and microbial activity was normalized to the wet weight of intestinal contents and expressed as A490 per gram of intestinal content [29].

#### 2.5.10. Intestinal Enzyme Activity Assay

The supernatant collected during the microbial activity assay was used as the crude enzyme solution for subsequent enzymatic activity measurements. Sucrase activity was measured at 540 nm using the 3,5-dinitrosalicylic acid (DNS) method, and protease activity was measured at 660 nm using the Folin–phenol assay, following established protocols. Each sample was assayed in triplicate. Enzyme activity was defined as one unit (U), corresponding to the amount of enzyme in 1 g of intestinal content that catalyzes the formation of 1 mg of product under specified conditions within a fixed reaction time, as previously described [30].

#### 2.5.11. High-Throughput Sequencing of the 16S rRNA Gene

For 16S rRNA sequencing, four biological samples per group were included after quality control. Alpha-diversity indices, including Chao1, Observed-species, Pielou_e, and Faith_pd, were calculated using QIIME2. Differences in alpha-diversity indices between groups were assessed using the Wilcoxon rank-sum test. Beta diversity was calculated based on Bray–Curtis distances and visualized using principal coordinate analysis (PCoA) and non-metric multidimensional scaling (NMDS). Differentially abundant taxa were identified using LEfSe analysis with an LDA score threshold of 2.0. PICRUSt2 was used only for exploratory prediction of microbial functional potential.

(1) Sample Collection: The small intestine was aseptically removed. After the luminal contents were expelled with sterile forceps, the intestinal tissue was longitudinally incised with sterile scissors. The intestinal wall was rinsed with sterile saline, blotted dry, and the mucosa was scraped using sterile cover slips. The mucosal scrapings from each mouse were placed in individual 1.5 mL sterile microcentrifuge tubes, flash-frozen in liquid nitrogen, and stored at −80 °C. The samples were sent to Shanghai Personal Biotechnology Co., Ltd. (Shanghai, China) for analysis of gut microbial structure and diversity via high-throughput 16S rRNA gene sequencing [31].

(2) Sequencing Data Processing and Quality Assessment: Raw sequence data underwent quality control that included modification, trimming, and the removal of low-quality reads. After this, denoising was performed to minimize sequencing errors. Denoised reads were then merged into complete sequences. Following merging, the DADA2 algorithm was applied to generate distinct amplicon sequence variants (ASVs). Quality of the processed data was assessed using dilution curves, species accumulation curves, and Good’s coverage index.

(3) Diversity Analysis: Alpha diversity, which describes the number of species and their evenness of distribution, was measured using the ASV table in QIIME2. This included indices such as Chao1 (which estimates species richness), Observed-species, Pielou_e (which measures evenness), and Faith_pd (which considers species’ evolutionary relationships). Beta diversity looks at how species differ between samples. Differences were measured with the Bray–Curtis method. Patterns in these differences were shown using two methods: Principal Coordinates Analysis (PCoA) and Non-metric Multidimensional Scaling (NMDS).

(4) Taxonomic Composition Analysis: ASV clustering and taxonomic classification produced a table with the composition and abundance of taxa at the family and genus levels for each sample. Bar charts visualized and compared the relative abundances of different taxa across samples.

(5) Feature Microbiota Analysis: LEfSe was employed to identify taxonomic units that were differentially abundant between groups. This method displays the taxonomic hierarchy of marker species that are significantly enriched within each group, highlighting their importance.

(6) Functional Prediction Analysis: The metabolic capacity of the microbiota was predicted using PICRUSt2. This tool creates groups based on function. These groups were compared with the KEGG database. This process estimated and counted the presence of different metabolic pathways.

#### 2.5.12. Correlation Analysis Method

The relationship between the mouse gut mucosal microbiota and mitochondrial respiratory chain complexes, ATP content, and IgG content was analyzed by calculating correlation coefficients. This determined whether these factors were significantly associated with the gut microbiota.

Spearman correlation and Redundancy Analysis (RDA) were used to explore associations among microbial taxa and physiological indicators; these analyses do not indicate causality.

### 2.6. Statistical Analysis and Figure Production

Statistical analysis was performed with IBM SPSS Statistics (v25.0). Data are presented as mean ± standard error of the mean (M ± SEM).

An independent-samples *t*-test was used when data were normally distributed, and variances were homogeneous. If the data were normally distributed but the variances were not homogeneous, a Welch’s *t*-test was used. When data were not normally distributed, the non-parametric Mann–Whitney U test was used. A value of *p* < 0.05 was considered statistically significant. A value of *p* < 0.01 was considered highly significant. Figures were generated using GraphPad Prism (v9.5) and Adobe Illustrator (v2024).(Significance levels: * *p* < 0.05, ** *p* < 0.01, *** *p* < 0.001, **** *p* < 0.0001).

## 3. Results

### 3.1. Alterations in the General Condition of Mice

**Figure 1:**
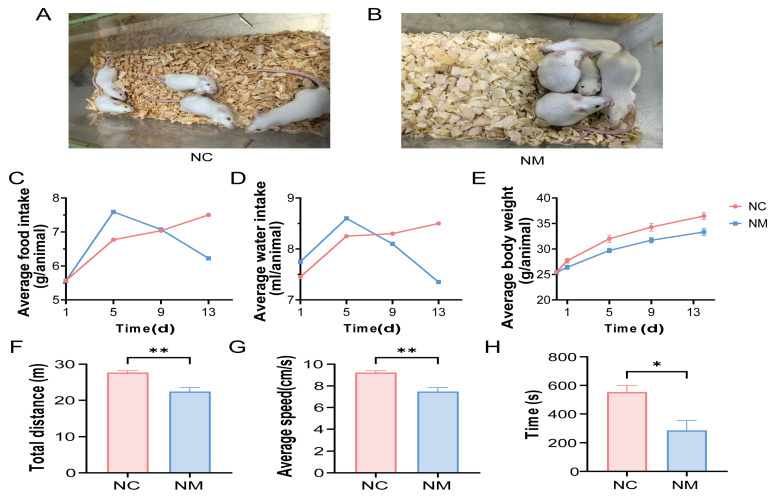
General Condition of Mice. (**A**) The general state of NC; (**B**) the general state of NM; (**C**) the average daily food intake; (**D**) the average daily water intake; (**E**) the average body weight; (**F**) the total movement distance; (**G**) the average speed; (**H**) the time of exercise. Data are presented as M ± SEM. Average food and water intake were recorded at the cage level and presented as descriptive trends; body weight n = 10 mice/group; open-field and rotarod n = 5 mice/group. * *p* < 0.05, ** *p* < 0.01. NC: normal control group; NM: fatigue model group.

At the end of the modelling period, mice in the NC exhibited higher activity levels and had smooth, glossy fur (Figure 1A). In contrast, mice in the NM showed reduced activity and tended to curl up in the corners of the cage (Figure 1B). Moreover, compared with NC mice, NM mice showed lower daily food and water intake, accompanied by a slower increase in body weight (Figure 1C–E).

We further conducted behavioral assessments. As shown in Figure 1F,G, the open-field test revealed that the total distance travelled and average speed of NM mice were significantly lower than those of the NC (*p* = 0.0034). The rotarod fatigue test showed that the NM mice had a significantly shorter exercise duration than the NC (Figure 1H, *p* = 0.0125). Collectively, these quantitative physiological and behavioral results supported the successful establishment of the fatigue mouse model.

### 3.2. Alterations in Intestinal Morphology and Intestinal Enzyme Activity of Mice

**Figure 2:**
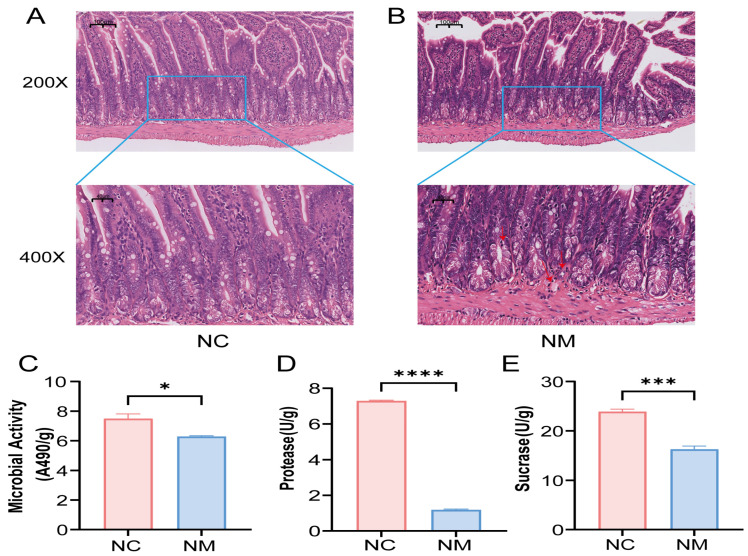
Intestinal Morphology and Intestinal Enzyme Activity of Mice. (**A**) The small intestinal tissue from NC; (**B**) the small intestinal tissue from NM; (**C**) microbial activity; (**D**) protease activity; (**E**) sucrase activity. Red arrows indicate mild inflammatory cell infiltration. Data are presented as M ± SEM (n = 3), and statistical significance was evaluated using an independent-samples *t*-test. * *p* < 0.05, *** *p* < 0.001, **** *p* < 0.0001. NC: normal control group; NM: fatigue model group.

As shown in Figure 2A,B, the H&E staining revealed that the small intestinal structure in the NC was clear and intact, with no significant oedema, inflammation, or lymphocyte infiltration in the intestinal mucosa. In contrast, mice in the NM showed disorganized intestinal villi, partial discontinuity of villus structures, and mild inflammatory cell infiltration in the crypts and muscular layer, as indicated by the red arrows. Based on these morphological observations, fatigue may be associated with alterations in intestinal tissue morphology in mice.

Consistent with these morphological observations, intestinal microbial activity was significantly lower in the NM than in the NC (Figure 2C, *p* = 0.0214). Additionally, intestinal enzyme activity analysis showed that protease activity was extremely significantly reduced (*p* < 0.0001), while sucrase activity was significantly reduced (*p* = 0.0007) in the NM (Figure 2D,E). Protease and sucrase are involved in the digestion of proteins and carbohydrates, respectively; therefore, reduced activities of these enzymes may impair the availability of key nutrient substrates, such as amino acids and glucose.

Together, these findings suggest that fatigue was associated with representative morphological alterations in the small intestine, accompanied by significant reductions in intestinal microbial activity and digestive enzyme function.

### 3.3. Alterations in Immune Status of Mice

**Figure 3:**
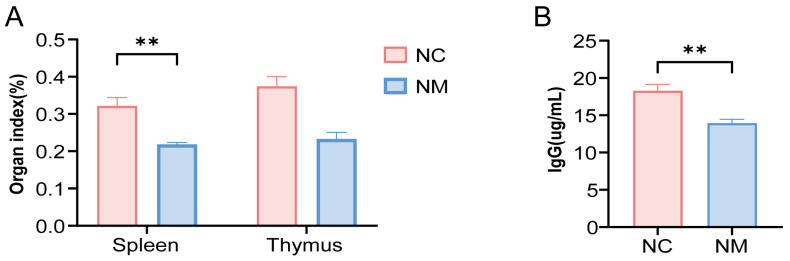
Immune Status of Mice. (**A**) Organ index; (**B**) IgG content. Data are presented as M ± SEM (n = 5), and statistical significance was evaluated using an independent-samples *t*-test. ** *p* < 0.01. NC: normal control group; NM: fatigue model group.

Compared with the NC, the thymus index in the NM showed a decreasing trend. However, this difference was not statistically significant. The spleen index was significantly decreased (Figure 3A, *p* = 0.0023). Serum IgG levels were also measured and were significantly lower in the NM than in the NC (Figure 3B, *p* = 0.0025). These findings indicate that fatigue was associated with immune-related indicators in mice.

### 3.4. Alterations in Energy Metabolism of Mice

**Figure 4:**
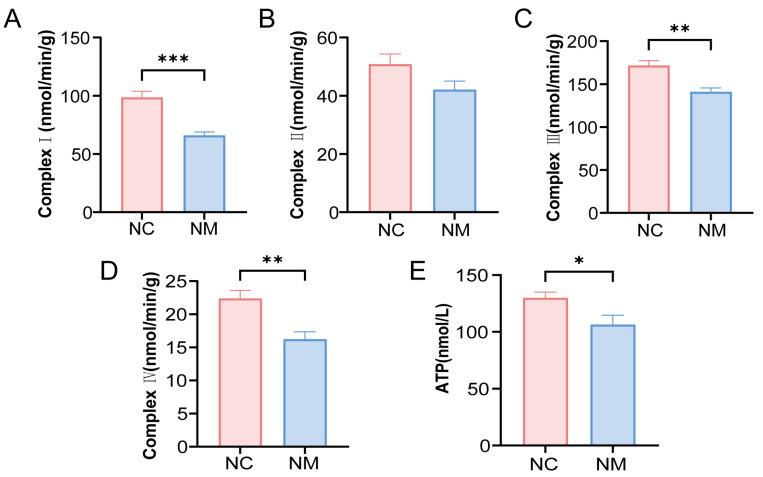
Alterations in energy metabolism of mice. (**A**) Activity of mitochondrial respiratory chain complex I; (**B**) activity of mitochondrial respiratory chain complex II; (**C**) activity of mitochondrial respiratory chain complex III; (**D**) activity of mitochondrial respiratory chain complex IV; (**E**) ATP content. Data are presented as M ± SEM (n = 5), and statistical significance was evaluated using an independent-samples *t*-test. * *p* < 0.05, ** *p* < 0.01, *** *p* < 0.001. NC: normal control group; NM: fatigue model group.

In comparison with the NC, the activities of mitochondrial respiratory chain complexes I (*p =* 0.0007), III (*p =* 0.0032), and IV (*p =* 0.0061) were significantly reduced in the NM (Figure 4A,C,D). The activity of complex II also decreased, but this change did not reach statistical significance (Figure 4B). Additionally, ATP content was significantly lower in the NM than in the NC (Figure 4E, *p =* 0.043). These findings suggest that the fatigue was associated with decreased mitochondrial energy-related indicators in thigh muscle.

### 3.5. Alterations in the Gut Microbiota of Mice

#### 3.5.1. Alterations in Gut Mucosal Microbiota Diversity of Mice

**Figure 5:**
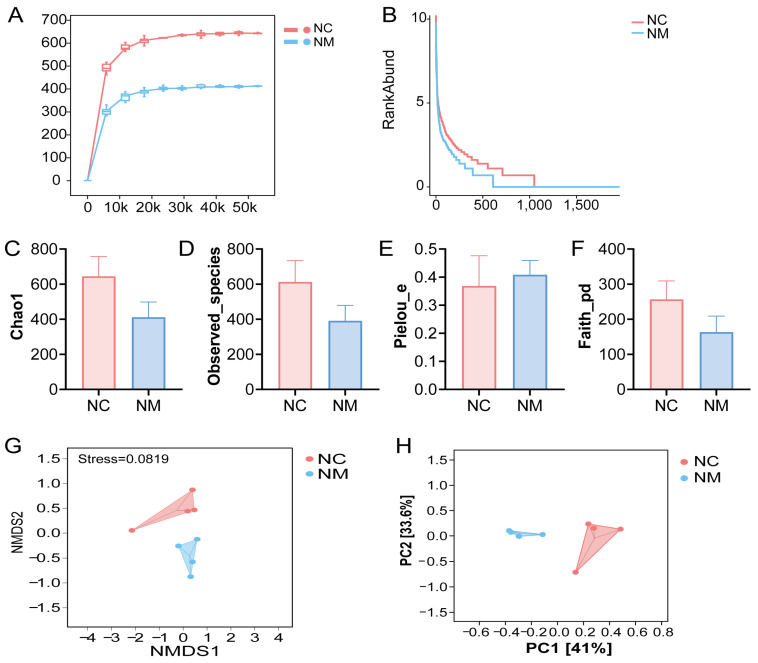
Changes in gut microbiota diversity of mice. (**A**) Rarefaction curve; (**B**) rank abundance curve; (**C**) Chao1 index; (**D**) Observed-species index; (**E**) Pielou_e index; (**F**) Faith_pd index; (**G**) NMDS analysis; (**H**) PCoA analysis. Alpha-diversity indices are presented as M ± SEM; beta diversity was visualized using NMDS and PCoA based on Bray–Curtis distances, n = 4. NC: normal control group; NM: fatigue model group.

For Alpha diversity, the Chao1 and Observed-species indices reflect microbial richness, the Pielou_e index reflects evenness, and the Faith_pd index reflects phylogenetic breadth. As depicted in Figure 5C–F, the NM exhibited a downward trend in the Chao1, Observed-species, and Faith_pd indices, while the Pielou_e index showed an upward trend. The rarefaction curve (Figure 5A), which compares the alpha diversity of different samples at an equal sequencing depth, indicates greater diversity in the NC. The rank abundance curve (Figure 5B), which reflects the distribution of ASV abundance across groups, shows higher abundance in the NC.

In terms of Beta diversity, NMDS analysis revealed a clear separation between the NC and NM (Stress = 0.0819; Figure 5G), confirming the reliability of the results. PCoA analysis showed that PC1 and PC2 explained 41% and 33.6% of the variance, respectively (Figure 5H). Collectively, these findings suggest that the fatigue model was associated with trends toward altered small-intestinal microbiota diversity in mice, although the limited sequencing sample size should be considered.

#### 3.5.2. Alterations in the Species Composition of the Gut Microbiota of Mice

**Figure 6:**
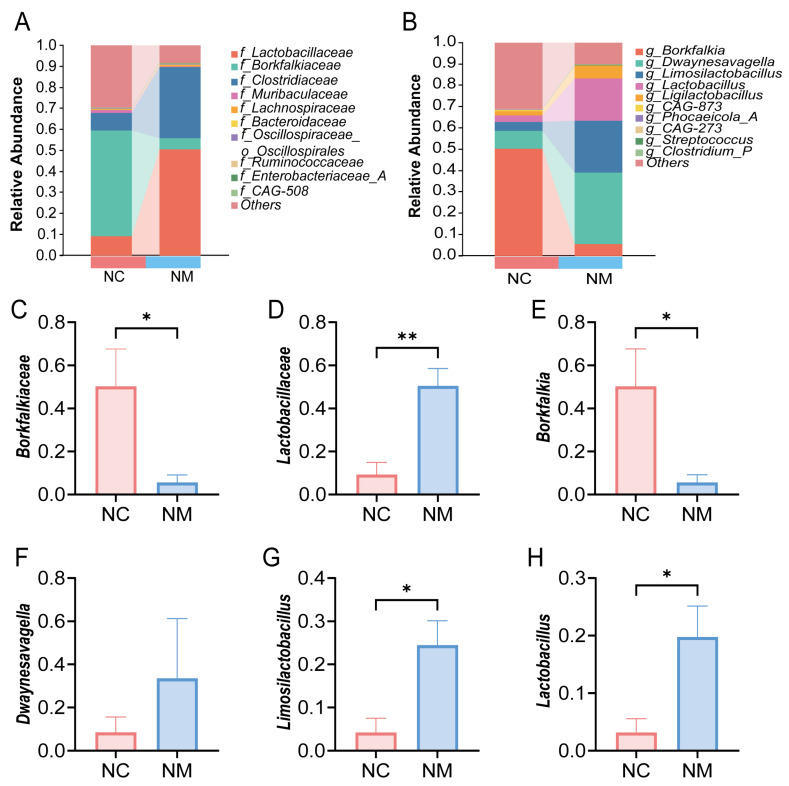
Alterations in the species composition of the gut microbiota. (**A**) The relative abundance plot at the family level; (**B**) the relative abundance plot at the genus level; (**C**) *Borkfalkiaceae*; (**D**) *Lactobacillaceae*; (**E**) *Borkfalkia*; (**F**) *Dwaynesavagella*; (**G**) *Limosilactobacillus*; (**H**) *Lactobacillus*. Data are presented as M ± SEM (n = 4), and statistical significance was evaluated using an independent-samples *t*-test. * *p* < 0.05, ** *p* < 0.01. NC: normal control group; NM: fatigue model group.

We quantified the relative abundances of the top ten dominant bacterial families and genera. At the family level, both groups were predominantly composed of *Lactobacillaceae*, *Borkfalkiaceae*, and *Clostridiaceae* (Figure 6A). Compared with the NC, the NM showed a significant decrease in *Borkfalkiaceae* abundance (Figure 6C, *p =* 0.0457), a significant increase in *Lactobacillaceae* abundance (Figure 6D, *p =* 0.0062), and a trend of increasing *Clostridiaceae* abundance.

At the genus level, the dominant genera in both groups were *Borkfalkia*, *Dwaynesavagella*, *Limosilactobacillus*, and *Ligilactobacillus* (Figure 6B). Relative to the NC, the NM exhibited a significant decrease in *Borkfalkia* abundance (Figure 6E, *p =* 0.0458), a trend of increasing *Dwaynesavagella* abundance (Figure 6F), and significant increases in the abundances of *Limosilactobacillus* (*p =* 0.0222) and *Lactobacillus* (Figure 6G,H, *p =* 0.0312).

#### 3.5.3. Alterations in Characteristic Gut Mucosal Microbiota of Mice

**Figure 7:**
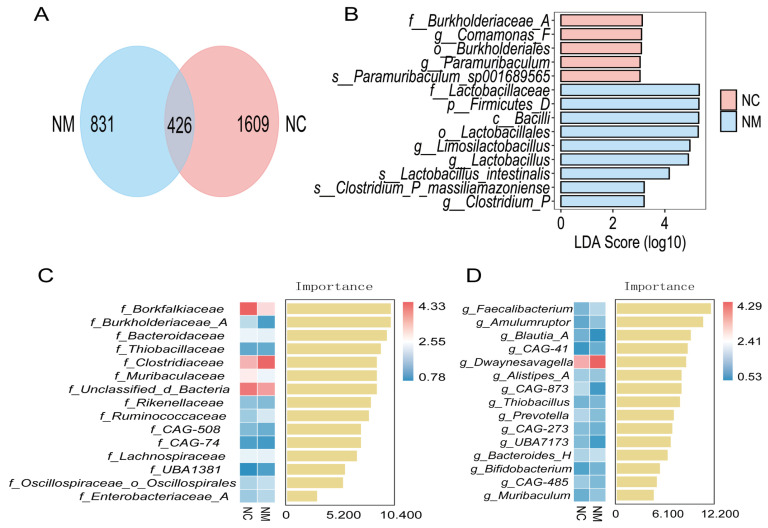
Characteristic Gut Mucosal Microbiota. (**A**) Venn diagram of ASVs; (**B**) LDA score histogram; (**C**) random forest analysis at the family level; (**D**) random forest analysis at the genus level. LDA score threshold = 2.0. NC: normal control group; NM: fatigue model group.

A Venn analysis revealed that the NC possessed 1609 unique ASVs, while the fatigue NM contained 831 unique ASVs, with 426 ASVs being common to both (Figure 7A).

This finding is consistent with the results from the species abundance rank curves. To identify differentially abundant taxa, an LDA effect size (LEfSe) was conducted, using an LDA score threshold of 2 (Figure 7B). The NC was characterized by the bacterial families *Burkholderiaceae-A*, *Comamonas-F*, and orders *Burkholderiales*, as well as the genus *Paramuribaculum*. In contrast, the NM was characterized by the family *Lactobacillaceae*, phylum *Firmicutes_D*, class *Bacilli*, order *Lactobacillales*, and genus *Limosilactobacillus*.

Further random forest analysis at the family level identified *Bacteroidaceae*, *Burkholderiaceae_A*, *Thiobacillaceae*, *Clostridiaceae*, and *Muribaculaceae* as the most significant features distinguishing the groups (Figure 7C). At the genus level, *Faecalibacterium*, *Amulumruptor*, *Blautia_A*, *CAG-41, Dwaynesavagella*, and *Alistipes_A* were identified as key discriminative features (Figure 7D).

#### 3.5.4. Alterations in Predicted Gut Microbiota Functions of Mice

**Figure 8:**
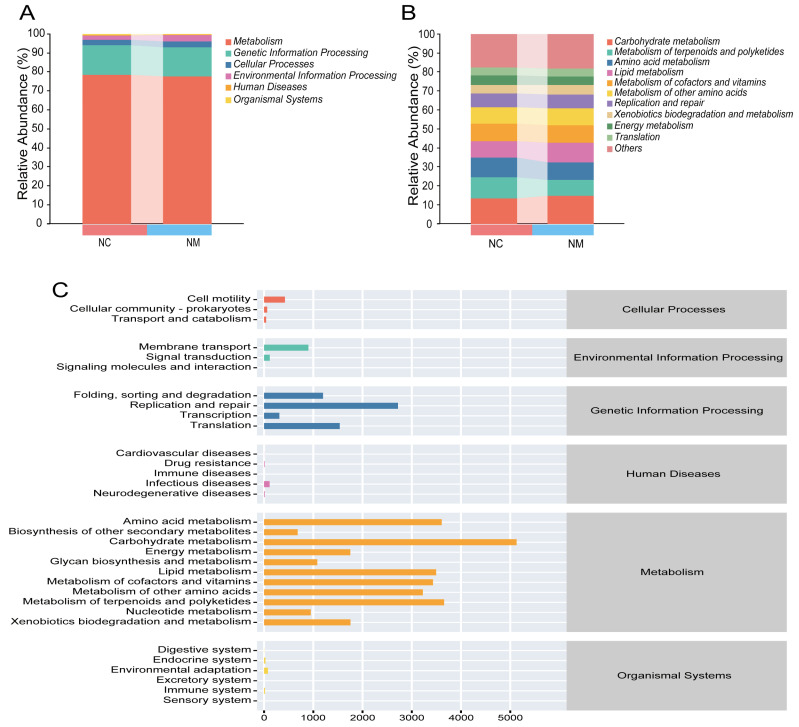
PICRUSt2-based functional prediction of gut microbiota. (**A**) Predicted KEGG functional categories at Level 1. (**B**) Predicted KEGG functional categories at Level 2. (**C**) Comparison of Level 1 and Level 2 pathways in functional prediction. These profiles represent predicted functional potential rather than directly measured metabolic activity. NC: normal control group; NM: fatigue model group.

Functional potential prediction, based on PICRUSt2, was utilized to assess the metabolic capabilities of the gut microbiota across different groups. KEGG pathway analysis at Level 1 revealed that the predicted functions were primarily focused on Metabolism, Genetic Information Processing, Cellular Processes, and Environmental Information Processing (Figure 8A). Notably, Metabolism was the most abundant category, underscoring the gut microbiota’s significant role in host metabolic regulation. When comparing the groups, the NM exhibited a slight decrease in the relative abundance of Metabolism pathways, whereas there was a concomitant increase in the relative abundance of Environmental Information Processing and Cellular Processes pathways.

KEGG analysis at Level 2 indicated that metabolism-related pathways, including Carbohydrate metabolism, Metabolism of terpenoids and polyketides, Amino acid metabolism, and Lipid metabolism, constituted the major functional components (Figure 8B). Specifically, the NC showed higher abundances in *Carbohydrate metabolism* and *Metabolism of terpenoids and polyketides*, while the NM demonstrated higher abundances in *Carbohydrate metabolism* and *Lipid metabolism*. A subsequent bar chart analysis (Figure 8C) confirmed that the primary differences were concentrated within the overarching Metabolism category, with the Level 2 pathways for *Carbohydrate metabolism*, *Metabolism of terpenoids and polyketides*, *Amino acid metabolism*, and *Lipid metabolism* all being subcategories of the Level 1 *Metabolism* pathway.

In conclusion, the PICRUSt2 functional prediction results suggest that fatigue may contribute to host metabolic dysfunction and physiological abnormalities by modulating gut microbiota metabolic functions, particularly through alterations in *Carbohydrate* and *amino acid metabolism*. However, these predictions should be interpreted cautiously because they were inferred from 16S rRNA gene sequencing data rather than directly measured metabolic activity.

#### 3.5.5. Correlation Analysis

**Figure 9:**
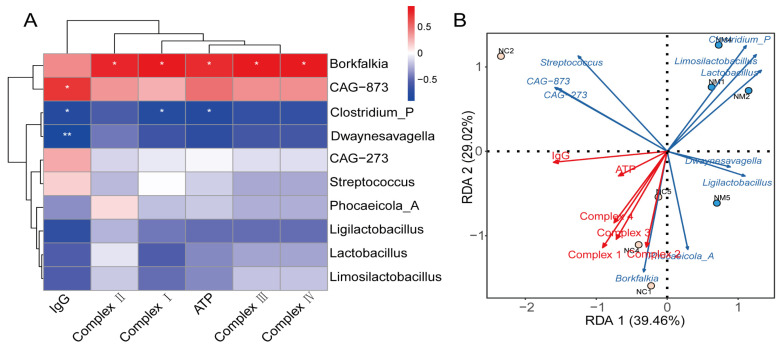
Correlation Analysis. (**A**) The correlation between genera and environmental factors. (**B**) the RDA plot at the genus level. Spearman correlation and RDA were used to explore associations; n = 4. * *p* < 0.05, ** *p* < 0.01; NC: normal control group; NM: fatigue model group.

To investigate the relationship between fatigue-related factors and the gut microbiota, Spearman’s correlation analysis was performed on the top 10 species ranked by total abundance. At the genus level (Figure 9A), *Borkfalkia* exhibited a significant positive correlation with mitochondrial Complexes I, II, III, IV, and ATP (*p* < 0.05). *CAG-873* showed a significant positive correlation with IgG (*p* < 0.05) and a positive trend with the mitochondrial complexes and ATP. Conversely, *Clostridium_P* was significantly negatively correlated with IgG, Complex I, and ATP (*p* < 0.05). *Dwaynesavagella* was highly significantly negatively correlated with IgG (*p* < 0.01) and showed a negative trend with the mitochondrial complexes and ATP.

The RDA results, depicted in Figure 9B, show that RDA1 and RDA2 explained 39.46% and 29.02% of the community variance, respectively. The mitochondrial respiratory chain complexes (Complex I–IV) and ATP vectors pointed in a consistent direction in the ordination plot, indicating association in the regulation of energy metabolism. IgG, however, occupied a relatively independent direction. The distribution of the samples revealed that the NC was primarily associated with the energy metabolism axis, while the NM clustered towards the microbial community space. This suggests that alterations in gut microbiota structure during fatigue are closely linked to dysregulation of energy metabolism. Regarding microbial distribution, *Phocaeicola_A* aligned with the mitochondrial function axis, suggesting a potential association with energy metabolism. Conversely, *Streptococcus*, *CAG-873*, and *CAG-273* were positioned in the opposite direction, implying an opposite direction with energy metabolism. *Clostridium_P*, *Lactobacillus*, *Limosilactobacillus*, and *Ligilactobacillus* were closely associated with the NM distribution, suggesting a potential connection to the fatigued state.

In conclusion, the Spearman correlation and RDA analyses consistently demonstrate a close link among the gut microbiota, the host’s mitochondrial energy metabolism, and immune function.

## 4. Discussion

### 4.1. Effects of Fatigue on Mitochondrial Energy Metabolism in Mice

Mitochondria serve as the central hub for cellular energy metabolism, providing essential energy support to the organism [32]. In this study, the fatigued mice exhibited significant reductions in the activities of mitochondrial respiratory chain complexes I, III, and IV in the thigh muscle, accompanied by a marked decrease in ATP content. These findings suggest that skeletal muscle was associated with reduced mitochondrial energy-related indicators under fatigued conditions.

The mitochondrial respiratory chain complexes I–IV constitute the core machinery for electron transport and ATP synthesis. Complexes I and II receive electrons from NADH and FADH_2_/succinate pathways, respectively, transferring them to coenzyme Q. Complex III subsequently transfers electrons to cytochrome c, and complex IV, as the terminal oxidase, reduces molecular oxygen to water [33,34]. This process involves two major electron transport routes: electrons derived from NADH are transferred through complexes I, III, and IV, whereas electrons derived from succinate enter the respiratory chain through complex II and are subsequently transferred through complexes III and IV [35,36]. The concurrent decrease in the activities of complexes I–IV observed in this study suggests that fatigue may suppress the overall function of the electron transport chain rather than affecting individual complexes in isolation.

Previous studies indicate that fatigue-associated mitochondrial dysfunction may interact with oxidative stress and inflammatory responses [37,38]. Muluye demonstrated that forced swimming-induced chronic fatigue in mice resulted in structural mitochondrial damage in skeletal muscle, accompanied by increased ROS-mediated lipid peroxidation, elevated TNF-α levels, and disrupted mitochondrial dynamics [39]. These findings imply that prolonged physical stress or fatigue may impair the mitochondrial inner membrane and respiratory chain function through oxidative stress and inflammation, thereby reducing oxidative phosphorylation and ATP production. Accordingly, mitochondrial dysfunction is considered a key biological mechanism underlying fatigue [40].

In conclusion, this study indicates that fatigue is associated with alterations in mitochondrial energy-related indicators, potentially contributing to insufficient energy supply through suppressed respiratory chain complex activity and reduced ATP production. Nevertheless, these observations do not constitute comprehensive evidence of mitochondrial dysfunction, and future investigations incorporating oxygen consumption rate measurements, mitochondrial membrane potential assessment, ROS quantification, and mitochondrial biogenesis markers are warranted to substantiate these results.

### 4.2. Effects of Fatigue on Immune Function in Mice

The thymus index and spleen index are metrics used to assess the status of immune organs and immune function [41]. In this study, fatigue model mice exhibited a downward trend in thymus index, while spleen index and serum IgG levels were markedly decreased, suggesting compromised immune-related parameters under fatigued conditions.

The thymus and spleen are pivotal immune organs. The thymus, as a central lymphoid organ, is responsible for T lymphocyte development and maturation, establishment of immune tolerance, and secretion of thymic hormones [42,43]. A reduction in thymus index typically indicates thymic atrophy, diminished T-cell production, and impaired cellular immunity. The spleen serves as a key site for antigen recognition and lymphocyte proliferation, regulating both humoral and cellular immune responses [44]. A decrease in the spleen index generally indicates splenic atrophy, reduced lymphocyte numbers, and a decline in the overall humoral and cellular immune capacity of the organism [45]. IgG is a core molecule of humoral immunity, responsible for recognizing and eliminating pathogens [46]. Therefore, reductions in thymus and spleen indices along with decreased IgG levels suggest that fatigue may compromise both cellular and humoral immunity.

Previous research has confirmed that immune function is abnormal in a state of fatigue [47], including reductions in the indices of major immune organs [48]. Moreover, fatigue or prolonged high-intensity exercise can impair immune cell function, reduce mucosal and humoral immunity, and increase susceptibility to infection [49].

Nonetheless, the immune assessments in this study were limited, as immune cell subsets, cytokine levels, mucosal immunity markers, and classical inflammatory indicators such as IL-6 and TNF-α were not measured.

### 4.3. Effects of Fatigue on the Murine Gut Microbiome

The gut microbiota constitutes a complex intestinal microbial ecosystem and serves as a critical regulator of host health [18]. Prior research has indicated that fatigue induces intestinal inflammation, compromises the intestinal mucosal barrier, and adversely affects the diversity and composition of the gut microbiota [50]. In this study, fatigue was associated with morphological alterations in the small intestine, reduced intestinal microbial activity, decreased protease and sucrase activities, and shifts in small-intestinal mucosal microbial composition. These findings indicate that fatigue may impair intestinal functional status and remodel the gut microbial community.

In the exploratory 16S rRNA gene sequencing analysis, the NM showed a decreasing trend in richness-related indices and alterations in dominant taxa. Compared with the NC, which was predominantly populated by *Borkfalkiaceae* and *Borkfalkia*, the NM exhibited a significant decrease in these bacteria. Instead, *Lactobacillaceae*, *Limosilactobacillus*, and *Lactobacillus* became the dominant taxa. The increase in *Lactobacillaceae* and *Limosilactobacillus* may reflect a compensatory or context-dependent microbial response to prolonged standing-induced physiological stress, which requires further validation.

KEGG pathway analysis revealed that *carbohydrate metabolism*, *amino acid metabolism*, and *lipid metabolism* were the predominant microbial pathways. Notably, *Paramuribaculum*, a common *Muribaculaceae*-related genus in the mouse intestine, is typically associated with complex carbohydrate degradation and maintenance of intestinal metabolic homeostasis [51]. Considering the observed reductions in digestive enzyme activities, fatigue may suppress host digestive and absorptive capacity and induce metabolic functional remodeling of the gut microbiota, particularly affecting carbohydrate and lipid metabolism.

In conclusion, fatigue may be associated with alterations in intestinal microecological homeostasis in mice, reducing microbial and digestive enzyme activities, and modifying microbial diversity and characteristic taxa. These changes may further influence nutrient utilization, energy supply, and immune-inflammatory homeostasis.

### 4.4. Associations Among Gut Microbiota, Energy Metabolism, and Immune-Related Indicators in Fatigued Mice

The gut microbiota is closely associated with host nutritional metabolism, intestinal barrier function, and immune homeostasis. Previous studies have suggested that gut microecology is closely linked to immune, metabolic, and neural functions and may be involved in the persistence of fatigue [52]. In addition, gut microbes can degrade dietary fibers and complex carbohydrates that are otherwise indigestible by the host, thereby producing short-chain fatty acids that contribute to energy supply and metabolic homeostasis [53]. In the present study, the prolonged standing-induced fatigue model was accompanied by alterations in gut microbiota composition, reduced intestinal microbial and digestive enzyme activities, decreased mitochondrial respiratory chain complex activities and ATP content, and lower serum IgG levels. These findings suggest that gut microbial alterations, mitochondrial energy-related changes, and immune-related changes may occur concurrently under this fatigued condition.

KEGG-based functional prediction indicated that the predicted microbial functions were mainly enriched in *metabolic pathways*, including *carbohydrate metabolism*, *amino acid metabolism*, and *lipid metabolism*. Together with the observed reductions in protease and sucrase activities, these results suggest that the fatigued state may be associated with altered intestinal digestive capacity and microbial metabolic potential. Spearman correlation analysis further showed that *Borkfalkia* was significantly positively correlated with mitochondrial respiratory chain complex I–IV activities and ATP content, whereas *Clostridium_P* and *Dwaynesavagella* were significantly negatively correlated with IgG levels. RDA also indicated that changes in the microbial community were associated with mitochondrial respiratory chain complex activities, ATP content, and IgG levels. These results support potential associations among gut microbiota composition, mitochondrial energy-related indicators, and immune-related parameters.

Therefore, this study suggests that fatigue may be associated with alterations in a gut microbiota–energy metabolism–immunity framework.

### 4.5. Limitations

This study has several limitations. First, no formal a priori sample size calculation was performed, and the sample size was determined based on previous related mouse studies, experimental feasibility, and ethical considerations. Second, the relatively small sample size, particularly for 16S rRNA gene sequencing, may reduce statistical power and increase uncertainty in diversity, differential abundance, functional prediction, and correlation analyses. Third, the present study does not establish causal relationships among gut microbiota alterations, mitochondrial energy-related indicators, and immune-related parameters. Finally, classical biomarkers related to stress, inflammation, metabolism, muscle injury, and oxidative damage, such as corticosterone, IL-6, TNF-α, lactate, creatine kinase, glucose, and oxidative stress-related indicators, were not assessed. Therefore, the present findings should be interpreted as associations among fatigue, intestinal microbial alterations, mitochondrial energy metabolism, and immune-related changes, rather than as definitive evidence of fatigue-specific mechanisms. Future studies with larger sample sizes, stress and inflammatory markers, mitochondrial functional assays, metabolomics, short-chain fatty acid profiling, and fecal microbiota transplantation are needed to clarify the biological significance and potential mechanisms underlying these associations.

Furthermore, this study was conducted in a mouse fatigue model, and the results can only serve as preliminary evidence for animal experiments. Human fatigue is influenced by multiple factors such as psychology, occupation and lifestyle. Mouse models cannot fully simulate human fatigue. Therefore, the results of animal experiments cannot be directly extrapolated to humans. However, the results of animal experiments can provide inspiration for everyone, and in the future, they can be further verified in human samples or clinically relevant fatigued populations.

## 5. Conclusions

This study reveals that fatigue was associated with reduced mitochondrial respiratory chain complex activities and ATP content, decreased immune-related indicators, impaired intestinal enzyme activities, and altered gut microbiota composition in mice. Correlation and RDA analyses indicated potential associations among gut microbiota structure, mitochondrial energy-related indicators, and serum IgG levels. These findings provide preliminary, association-based evidence for a potential gut microbiota–energy metabolism–immunity framework in fatigue-related physiological alterations. Although no nutritional intervention was performed, the examined indicators are closely related to nutritional physiology and may provide preliminary information for future nutrition-related mechanistic and intervention studies. Further studies incorporating dietary or functional food interventions are needed to determine the translational relevance of these findings.

## Data Availability

The gut microbiome sequencing data have been uploaded to the NCBI database (https://www.ncbi.nlm.nih.gov/), no. PRJNA1456534.
